# Effects of synbiotics on necrotizing enterocolitis and full enteral feeding in very low birth weight infants: A double-blind, randomized controlled trial

**DOI:** 10.1097/MD.0000000000039647

**Published:** 2024-09-13

**Authors:** Amir Ali Mahboobipour, Ali Bitaraf, Pourang Mohammadi, Mina Khosravifar, Homa Babaei, Amir Shahidolahi

**Affiliations:** a Tracheal Diseases Research Center, National Research Institute of Tuberculosis and Lung Diseases, Shahid Beheshti University of Medical Sciences, Tehran, Iran; b School of Medicine, Kermanshah University of Medical Sciences, Kermanshah, Iran; c Department of Pediatrics, Imam Reza Hospital, Kermanshah University of Medical Sciences, Kermanshah, Iran.

**Keywords:** clinical trial, feeding intolerance, necrotizing enterocolitis, premature infants, synbiotics

## Abstract

**Background:**

Necrotizing enterocolitis (NEC) is a multifactorial disease primarily affecting infants with very low birth weight (VLBW). Research has shown that the pathogenesis of NEC is associated with abnormal bacterial colonization. Synbiotics, dietary supplements containing probiotics (beneficial bacteria) and prebiotics (non-digestible food), can alter the gut microbiome.

**Methods:**

A double-blind, randomized controlled trial was conducted to assess the efficacy of PediLact^®^, an oral drop multi-strain synbiotic containing *Lactobacillus rhamnosus*, *Lactobacillus reuteri*, and *Bifidobacterium infantis*, on nutritional parameters and the occurrence of NEC in VLBW neonates. In this study, 118 VLBW neonates from neonatal intensive care units were randomly allocated in a 1:1 ratio to receive either a synbiotic or a placebo. The synbiotic administration continued until the infant was fully fed. The primary outcomes were the occurrence of NEC and the number of days required to achieve full enteral feeding. Log-binomial regression and ANOVA/ANCOVA models were used for analysis.

**Results:**

In the group that received the synbiotic, the incidence of NEC decreased significantly (adjusted RR = 0.22, 95% CI: 0.07–0.72, *P* value = .01; adjusted RD = −0.22, 95% CI: −0.33 to −0.12, *P* value < .001; NNT = 5). Additionally, feeding intolerance was less frequent in this group (adjusted RR = 0.27, 95% CI: 0.14–0.51, *P* value < .001; NNT = 3). Furthermore, consumption of the synbiotic was associated with significant weight gain (approximately 40 g) in infants (adjusted SMD = 0.63; 95% CI: 0.26–1.00, *P* value < .001). The duration of hospitalization and the time required to reach full enteral feeding were also significantly shorter in the synbiotic group (by approximately 3 days). No serious side effects were reported.

**Conclusion:**

Prescribing multi-strain synbiotics reduces the incidence of NEC in VLBW infants and has beneficial effects on breastfeeding tolerance and weight gain velocity. Therefore, physicians may consider prescribing synbiotics to VLBW neonates.

## 1. Introduction

Preterm or premature infants are characterized by gestational age of less than 37 weeks.^[1]^ These infants have a high risk of mortality and morbidity due to the immaturity of their organ systems, making them more prone to serious complications.^[1,2]^ After respiratory failure, inflammatory phenotypes observed in preterm infants, such as sepsis or necrotizing enterocolitis (NEC), are the second leading cause of death in preterm infants.^[2]^

NEC, defined as acute inflammatory necrosis of the gastrointestinal (GI) tract, primarily affects neonates with very low birth weight (VLBW) (weighing less than 1500 g).^[3,4]^ NEC has an incidence of 7% among VLBW infants and leads to death in 20% to 30% of cases. It is also associated with considerable morbidity, including neurodevelopmental impairment, resulting in a substantial burden.^[3]^

The exact pathogenesis of NEC, a multifactorial disease, is not fully understood.^[5]^ However, various risk factors are known to play a role in its development. One such risk factor is low birth weight, as microbiome formation in preterm infants is incomplete.^[6]^ In other words, the GI system of preterm infants is immature in some functions, such as digestion, motility, intestinal permeability, barrier defense function, immune defense, and anti-inflammatory effects.^[4]^ Moreover, preterm infants are more exposed to the hospital environment compared to mature infants, which promotes GI colonization with pathogenic bacteria.^[7]^ Studies have shown that the pathogenesis of NEC is strongly linked to abnormal bacterial colonization, leading to decreased GI diversity and altered bacterial strains with pathogenic bacteria.^[8–10]^

Bifidobacteria is the predominant bacteria in the GI tract of breastfed term infants.^[11]^ However, in preterm infants, Proteobacteria dominate, which may contribute to their susceptibility to NEC.^[12]^ Comparing the gut bacteria of preterm and term infants, preterm infants have higher amounts of Enterobacter, Enterococcus, Escherichia, and Klebsiella than term infants.^[7]^ In VLBW infants admitted to the neonatal intensive care unit (NICU), primarily due to respiratory problems, full breastfeeding cannot be initiated during the first few days after birth. For these infants, parenteral nutrition increases the likelihood of colonization by pathogenic bacteria in their intestines, elevating malnutrition risk and NEC.^[13]^

Feeding intolerance is a prevalent issue among neonates admitted to the NICU. It not only exacerbates morbidity and mortality in premature infants but also extends hospitalization time and can lead to NEC.^[14,15]^ Enteral feeding intolerance occurs in 27% of preterm infants and is characterized by an increase in the residual volume of the stomach, abdominal distention, and vomiting following enteral nutrition, which usually disrupts the patient’s feeding plan.^[15,16]^

Probiotics are beneficial bacteria that can improve the health of individuals when consumed in sufficient quantities. The colonization of the GI tract with probiotics (nonpathogenic bacteria) can competitively inhibit attachment by pathogenic bacteria, consequently decreasing the likelihood of NEC.^[17,18]^ Prebiotics are non-digestible nutrients host bacteria use to foster the growth of helpful microorganisms.^[19]^ Synbiotics are dietary supplements containing both probiotics and prebiotics. Synbiotics may benefit preterm infants by increasing mucosal barrier function, improving nutrition, regulating the immune system, and reducing mucosal colonization with intestinal pathogens.^[20–23]^

Several studies have been conducted on the effectiveness of different strains of probiotics in reducing the incidence of NEC in preterm infants. However, not all species of probiotics can reduce the incidence of NEC. Therefore, it is recommended to administer multi-strain probiotics.^[24]^ Regarding the effect of adding prebiotics, meta-analyses by the Cochrane group have shown that synbiotics may be more effective than probiotics in preventing severe NEC (82% vs 46% in the relative reduction of NEC incidence).^[25,26]^ However, the evidence had low certainty, and they suggested conducting high-quality clinical trials to demonstrate the actual effect of synbiotics.^[26]^

Based on these points, further clinical trial evidence is needed to determine the efficacy of synbiotics on GI health and prevent mortality and morbidity in preterm infants before making any recommendations for routine synbiotic application. In this study, we conducted a clinical trial to assess the efficacy of PediLact^®^, an oral drop multi-strain synbiotic containing *Lactobacillus rhamnosus*, *Lactobacillus reuteri*, and *Bifidobacterium infantis*, on nutritional parameters and the rate of NEC in VLBW infants.

## 2. Methods

### 2.1. Patients

This study was carried out on newborns with a very low birth weight of less than 1500 g and a gestational age of less than 34 weeks – early preterm – who were admitted to Imam Reza Hospital, Kermanshah, Iran. Neonates were excluded from the study by predetermined exclusion criteria, including gestational age ≥ 34 weeks, birth weight < 1000 g given the increased mortality or > 1500 g, age > 28 days since birth and presence of gastrointestinal obstruction, gastroschisis, congenital heart disease, sepsis or grade 2 or 3 asphyxia. This study also excluded neonates who were born to addicted mothers or those with a family history of immunodeficiency in first-degree relatives and who received infant formula or a synbiotic prior to admission.

### 2.2. Trial design and intervention

Assuming a type I error of 0.05 and a type II error of 0.2 and the occurrence of NEC being 3% in the intervention group and 18% in the control group, the sample size was estimated to be 64 neonates in each group.^[27]^ However, due to the study’s execution limitations, the study was conducted with 59 neonates in each group.

In this double-blind, parallel-group study, 118 neonates were randomly allocated in a 1:1 ratio to receive either a synbiotic or a placebo. Infants were randomized into the 2 treatment and placebo groups using a table of random numbers. Neonates with numbers from 0 to 4 were assigned to the intervention group, while those with numbers from 5 to 9 were assigned to the placebo group. The participants’ parents, the nurses who fed the neonates, and the residents who assessed outcomes were all blinded to the assignment and prescribed medications.

In the intervention group, after the infant’s milk volume reached 10 cc/kg/d, an oral synbiotic was administered at 1 drop/kg every 12 hours. The synbiotic administration continued until the infant was fully fed (150 cc/kg/d). In the control group, distilled water was given as a placebo after the infant’s milk volume reached 10 cc/kg/d. One drop/kg of body weight was administered every 12 hours until full feeding (150 cc/kg/d). In both groups, blinded NICU nurses administered drops, and if neonates tolerated the milk in their last meals, their daily milk volume would increase by 20 cc/kg. The milk used for feeding the infants was breast milk, which was nourished through a nasogastric tube.

The oral synbiotic (PediLact^®^) used in this study was manufactured by Zist Takhmir Pharmaceutical Company, located in the Islamic Republic of Iran. Each 0.05 cc (1 drop) oral synbiotic contained a probiotic compound (*Lactobacillus reuteri*, *Lactobacillus rhamnosus*, and *Bifidobacterium infantis* in the amount of 10^9^ colony forming units blend), prebiotic compounds including 3% fructooligosaccharides, and other ingredients including sunflower oil, medium-chain triglyceride, silicon dioxide, and natural flavoring. Additionally, the placebo was prepared in packages with the same shape and color as the synbiotic.

### 2.3. Trial oversight

This study was supported by the Kermanshah University of Medical Sciences (KUMS) and followed the International Conference on Harmonization guidelines for Good Clinical Practice and ethical principles of the Declaration of Helsinki. Informed consent was obtained from infants’ parents before participation. The study protocol was registered and approved by the Research Ethics Committee of KUMS (registry code: IRCT20180519039715N2, ethic code: IR.KUMS.REC.1396.702).

### 2.4. Endpoints and assessments

In this study, the effectiveness of synbiotics in treating neonates was evaluated based on nutritional parameters and the prevention of necrotizing enterocolitis (NEC). The primary outcomes included the occurrence of NEC, at any stage (even stage one) using modified Bell’s staging criteria, and the duration until achieving full enteral feeding (defined as sustaining 150 cc/kg/d for 24 hours).^[28]^ The final diagnosis of NEC was determined by neonatal specialists who were blinded to the study, ensuring impartial assessment. Secondary outcomes focused on feeding intolerance, discharge weight, and hospital stay length. Neonates experiencing emesis, abdominal distention, or significant gastric residuals (exceeding 20% of the previous feeding) were categorized as having feeding intolerance. Data collection was meticulously conducted using structured checklists throughout the study period. Baseline information, including demographic details (gender, gestational age, birth weight) and daily neonatal assessments, was recorded. Apart from NEC diagnosis, daily assessments were performed by trained nurses and residents who were unaware of the treatment allocation, ensuring objective evaluation of outcomes.

### 2.5. Statistical analysis

IBM SPSS Statistics (version 22.0, SPSS, Inc., Chicago) and Stata (version 14.2, Texas) software are used for data entry and analysis, respectively. Categorical variables are expressed as numbers (percentages), and continuous variables are reported as median [IQR] or mean (SD) as appropriate. The demographic variables with considerable differences between the study’s groups are considered for multivariate analysis.^[29]^ Then, if they change the effect size by at least 10%, they are kept in the final model. The log-binomial regression model is used for the categorical outcome (NEC occurrence) analysis. Risk ratio (RR), risk difference (RD), and number needed to treat (NNT) are reported as effect sizes as well (trivial: RD > −0.06, small: −0.14 < RD ≤ −0.06, moderate: −0.21 < RD ≤ −0.14, large: RD ≤ −0.21).^[30]^ Analysis of Variance/Covariance (ANOVA-ANCOVA) measures continuous outcomes and controls potential covariates or confounders. The equality of variances between the groups is tested using Levene’s test. Mean difference (MD) and standardized mean difference (SMD or Cohen’s d, SMD < 0.2 = trivial, 0.2 ≤ SMD < 0.5 = small, 0.5 ≤ SMD < 0.8 = moderate, SMD ≥ 0.8 = large) are used as models’ effect sizes.^[31]^ *P* < .05 is considered to be significant.

## 3. Results

**Figure 1.**
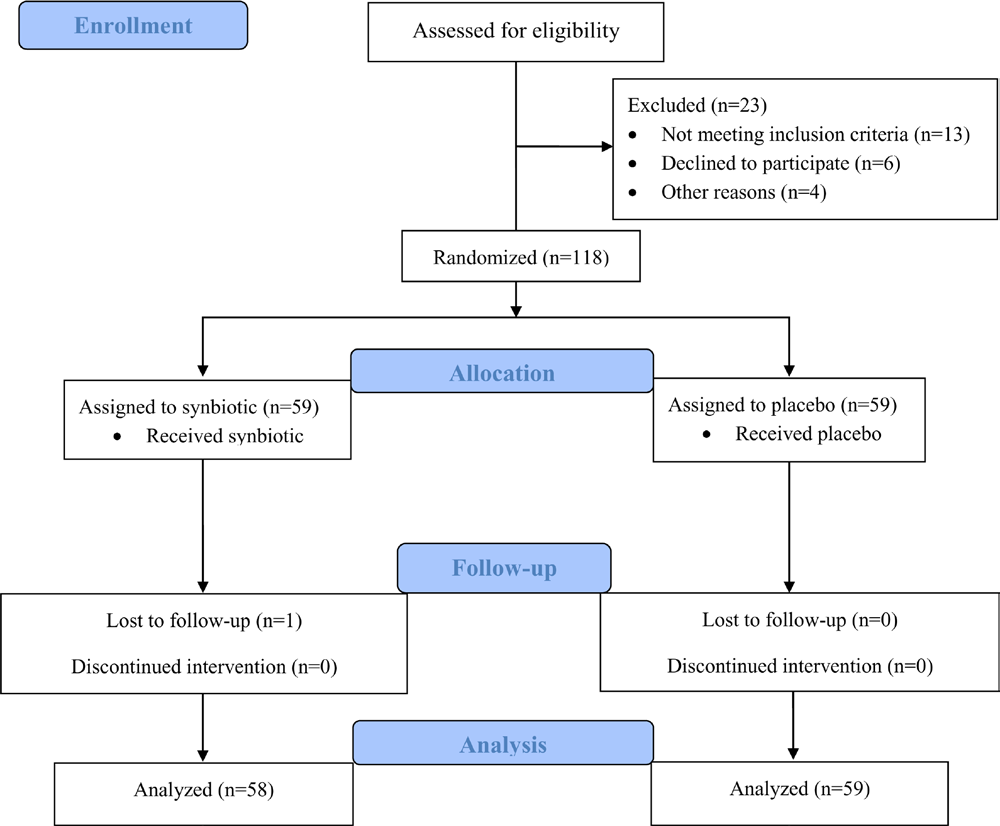
Consolidated standards of reporting trials (CONSORT) diagram.

In this study, from October 2018 to October 2019, 141 patients were screened, and 118 preterm neonates were included, with equal proportions in 2 groups (59 in each group). One group received the synbiotic, and the other received the placebo. All infants were monitored until the discharge date. Only 1 infant was lost to follow-up due to prematurity-related death (intraventricular hemorrhage) in the synbiotic group (Fig. 1).

Table 1 shows the demographic and clinical characteristics of the infants in each group. The number of boys in the intervention and control groups is 25 (43%) and 36 (61%), respectively. Also, the mean (SD) of newborns’ birth weight in the synbiotic and placebo groups are 1267.9 (148.6) and 1277.3 (146.0) grams, respectively. Birth weight and gestational age do not have a considerable difference between groups; however, there is a significant difference in sex and type of delivery between groups (more than a 10% difference) (Table 1).

**Table 1:** Baseline characteristics of participants.

Variables	Level	Placebo	Synbiotic	*P* value
		N = 59	N = 58	
Sex, N (%)	Male	36 (61)	25 (43)	.05*
	Female	23 (39)	33 (57)	
Type of delivery, N (%)	NVD	12 (20)	4 (7)	.03*
	C/S	47 (80)	54 (93)	
Birth weight (g), mean (SD)		1277.3 (146.0)	1267.9 (148.6)	.73†
Gestational age (wk), median (IQR)		31.0 (30.0-32.0)	31.0 (29.0-31.0)	.19†

C/S = cesarean section, NVD = natural vaginal delivery.

* Based on chi-square.

† Based on independent-samples *t* test

Table 2 demonstrates the NEC diagnosis based on the arms of the study. None of the infants developed stage III NEC. Stage II NEC occurred in 5 infants in the placebo group and 1 in the intervention group. Based on the log-binomial regression model, we calculated RR for NEC. The crude RR = 0.22 (95% CI: 0.07–0.72, *P* value = .01), and if we adjust RR for sex and type of delivery in multivariate analysis, adjusted RR = 0.22 (95% CI: 0.07–0.72, *P* value = .01). So, adjusted RR and crude RR are equal. Moreover, the adjusted difference between the risk of NEC in the synbiotic and placebo groups is −22% (adjusted RD = −0.22, 95% CI: −0.33 to −0.12, *P* value < .001). Hence, the NNT for NEC is 4.5 (95% CI: 3.0–8.3, *P* value < .001) (Table 2). The post hoc power analysis showed that the power of this study for the NEC outcome is 82%.

**Table 2:** The occurrence of necrotizing enterocolitis (NEC) in the synbiotic and placebo groups.

	Necrotizing enterocolitis +	Necrotizing enterocolitis −
Synbiotic	3 (5.2%)	55 (94.8%)
Placebo	14 (23.7%)	45 (76.3%)

Nine infants (15.5%) in the synbiotic group had feeding intolerance episodes during the study. In comparison, this statistic was 33 infants (55.9%) in the placebo group (adjusted RR for sex and type of delivery = 0.27, 95% CI: 0.14–0.51, *P* value < .001). There was about a 43% difference in adjusted absolute risk between groups. Therefore, the NNT for feeding intolerance is 2.3 (95% CI: 1.7–3.6, *P* value < .001).

**Figure 2.**
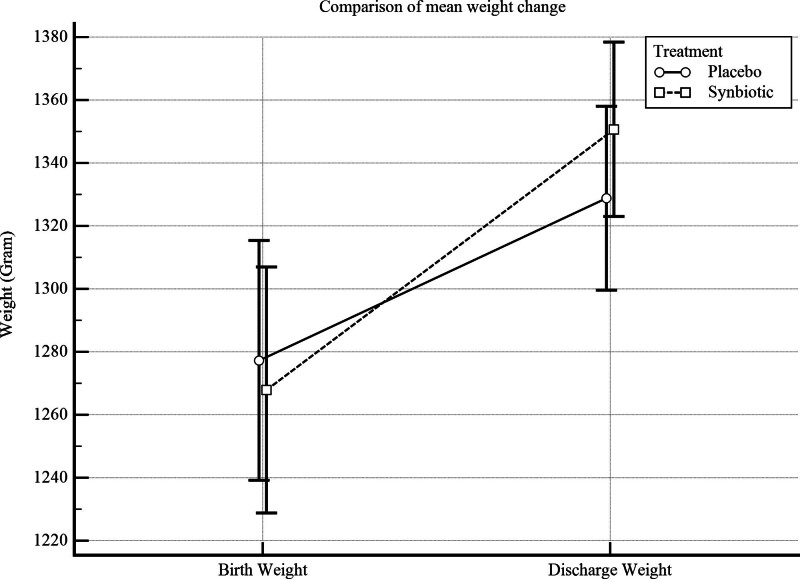
Weight changes in the synbiotic and placebo groups (error bar represents 95% CI for mean [±2 standard errors]).

In addition, Table 3 presents the effect of oral synbiotics on child weight at discharge by ANOVA/ANCOVA model analysis. As seen in multivariate analysis, neonates who received synbiotics had approximately 40 grams more weight at discharge from the hospital (adjusted MD = 39.9; 95% CI: 16.8–63.0, *P* value < .001). Also, adjusted Cohen’s d for weight difference at discharge time is about 0.6 (adjusted SMD = 0.63; 95% CI: 0.26–1.00, *P* value < .001) (Table 3 and Fig. 2).

**Table 3:** Discharge weight (gram) distribution in pre & post intervention by study groups in according to different models.

Model	Synbiotic (SD) n = 58	Placebo (SD) n = 59	Mean difference (95% CI)	Cohen’s d (95% CI)	*P* value*	Adjusted *R*^2^
Crude	1350.7 ± 105.3	1328.8 ± 112.2	21.9 (−18.0, 61.7)	0.20 (−0.16, 0.56)	.28	< 0.01
Adjusted†	1353.5 ± 65.7	1326.1 ± 65.7	27.4 (3.3, 51.5)	0.42 (0.05, 0.78)	.03	0.64
Adjusted‡	1359.8 ± 62.1	1319.9 ± 62.1	39.9 (16.8, 63.0)	0.63 (0.26, 1.00)	<.001	0.69

* Calculated for intervention based on ANOVA/ANCOVA model.

† Adjusted for baseline pretreatment weight (calculated based on ANOVA/ANCOVA model).

‡ Adjusted for baseline pretreatment weight and potential covariates (gender and delivery) (calculated based on ANOVA/ANCOVA model).

**Figure 3.**
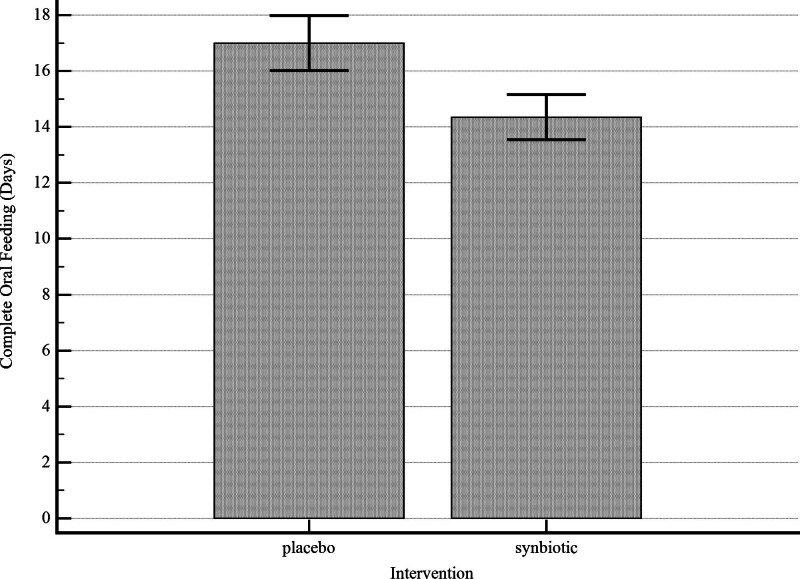
Full feed achievement in the synbiotic and placebo groups (error bar represents 95% CI for mean [±2 standard errors]).

The ANOVA/ANCOVA model was applied to determine the effect of oral synbiotics on time to reach full enteral feeding and duration of hospitalization in premature newborns. After adjusting for potential covariates in the multivariate analysis, a statistically significant association was found between oral synbiotic and reaching full enteral feeding (adjusted SMD = −0.98; 95% CI: −1.36 to −0.60, *P* value < .001) and duration of hospitalization (adjusted SMD = −0.58; 95% CI: −0.95 to −0.21, *P* value = .002). The average length of hospital stay and time to reach full enteral feeding in infants that received synbiotics is approximately 3 days less than the placebo group (adjusted MD = −3.0; 95% CI: −4.9 to −1.1, *P* value = .002; adjusted MD = −2.8; 95% CI: −3.8 to −1.7, *P* value < .001, respectively) (Table 4 and Fig. 3).

**Table 4:** Full feed achievement (days) and length of hospitalization (days) distribution by study groups in according to different models.

Outcome	Model	Synbiotic (SD) n = 58	Placebo (SD) n = 59	Mean difference (95% CI)	Cohen’s d (95% CI)	*P* value*	Adjusted *R*^2^
Full feed achievement	Crude	14.3 ± 3.1	17.0 ± 3.8	−2.7 (−3.9, −1.4)	−0.77 (−1.14, −0.39)	<.001	0.12
	Adjusted†	14.3 ± 2.8	17.1 ± 2.8	−2.8 (−3.8, −1.7)	−0.98 (−1.36, −0.60)	<.001	0.41
Length of hospitalization	Crude	24.9 ± 8.8	27.5 ± 9.0	−2.6 (−5.8, 0.7)	−0.29 (−0.65, 0.08)	.12	0.01
	Adjusted†	24.7 ± 5.2	27.7 ± 5.2	−3.0 (−4.9, −1.1)	−0.58 (−0.95, −0.21)	.002	0.66

* Calculated for intervention based on ANOVA/ANCOVA model.

† Adjusted for potential covariate (birth weight) (calculated based on ANOVA/ANCOVA model).

Regarding safety, all neonates survived, and no major complication due to intervention or immaturity occurred, except for one in the synbiotic group, who died on the fourth day of hospitalization due to intraventricular hemorrhage of the brain (more likely related to being premature than to the intervention).^[32]^ No serious side effects have been reported. Regarding NEC management, none of the infants required surgery.

## 4. Discussion

The present study aims to evaluate the efficacy of a synbiotic manufactured by Bio Fermentation Company from Iran (containing *Lactobacillus reuteri*, *Lactobacillus rhamnosus*, and *Bifidobacterium infantis*) on VLBW neonates. We assessed the synbiotic’s effectiveness from several aspects: NEC occurrence, parameters related to infants’ nutrition derived from breast milk (feeding tolerance, time to reach full enteral feeding, and weight gain), and duration of hospitalization.

In the present study, synbiotics decreased the risk of NEC of all stages by 78% in VLBW neonates compared to placebo. Moreover, the risk difference for NEC is 22% between the study groups, which is considered a large effect size. Therefore, the NNT for this purpose will be 5, which means that for every 5 neonates who received the synbiotic supplement, the synbiotic prevented NEC development in one of them. Generally, the number of RCTs investigating the effect of synbiotics on NEC in VLBW infants is relatively low.^[27,33–37]^ In Nandhini’s RCT, VLBW infants who received multi-strain synbiotics had about 50% lower occurrence of NEC in all stages compared to those who did not receive any intervention, but this was not statistically significant (7.4% vs 14.5%; *P* value = .18).^[33]^ Moreover, another RCT showed that administering multi-strain synbiotics is associated with an 80% relative risk reduction in the occurrence of NEC in preterm infants (*P* = .02).^[34]^ Furthermore, the results of an RCT by Dilli et al, which recruited 400 VLBW neonates, indicate that the combination of *Bifidobacterium lactis* and inulin as a synbiotic significantly decreased the NEC stage ≥ 2 rates (estimated RR = 0.22, 95% CI: 0.08–0.63; estimated RD = −0.14, 95% CI: −0.22 to −0.06).^[27]^ Finally, in a systematic review and meta-analysis conducted by the Cochrane group, they concluded that synbiotics might decrease the risk of stage II-III NEC in VLBW neonates (synbiotics compared to placebo: RR = 0.18, 95% CI: 0.09–0.40; RD = −0.07, 95% CI: −0.10 to −0.04).^[26]^

The results of different RCTs on mortality are inconsistent. Some RCTs suggest that synbiotics could decrease mortality in VLBW neonates, but some mention no evidence.^[27,33,35]^ We could not investigate this aspect in the present study because our mortality rate was much lower than that of similar RCTs (probably due to close monitoring).

In our study, synbiotics improved the nutritional parameters of preterm infants fed breast milk. Indeed, they reached full enteral feeding about 3 days earlier than the placebo group (adjusted SMD = −0.98). Moreover, for every 3 infants who received synbiotics, milk intolerance episodes were prevented in one of them (NNT = 3). Both effect sizes are large, which means the desirable impact of synbiotics. Also, synbiotics caused more adjusted weight gain in infants who received them (about 40 g, *P* value < .001). These findings show that adding synbiotics to the diet of VLBW infants could be an excellent option to enhance their nutritional parameters. However, these results should be interpreted cautiously since more evidence is needed. Güney-Varal et al showed a similar effect of synbiotics for feeding intolerance and time to reach full enteral feeding in VLBW neonates in an RCT. However, they did not investigate synbiotic efficacy on weight gain.^[35]^ In Dilli’s RCT, although synbiotics significantly decreased feeding intolerance (estimated RD = −0.05) and time to reach full enteral feeding (about 5 days) in VLBW neonates compared to placebo, it did not affect weight gain velocity.^[27]^ Underwood’s RCT also did not show the effectiveness of synbiotics on weight gain in premature infants.^[36]^ In contrast, another similar RCT demonstrated that synbiotic consumption significantly correlates with daily weight gain in premature babies.^[37]^ Therefore, there is inconsistency in articles, and systematic reviews and meta-analyses are needed to demonstrate the actual effect.

In this study, it was observed that the prescription of synbiotics is associated with a 3-day reduction in the duration of hospitalization in VLBW infants (adjusted SMD = −0.58). This effect is considered a moderate effect size. As a result of the decrease in hospitalization duration, there will also be a reduction in costs and hospital-acquired infections. However, similar RCTs have not shown that synbiotics could reduce hospital stays compared to a placebo, and this effect was found in our study.^[33–35,37]^ It may be due to the small number of similar studies and sample sizes since a Cochrane meta-analysis on the efficacy of probiotics in 5458 preterm infants shows that probiotics reduce hospital stay by 2 days on average (*P* value = .04).^[25]^

Our study had several limitations. First, we did not investigate the long-term effects of synbiotics. There are concerns about potential future side effects, which require several years of follow-up. Second, due to implementation limitations, our sample size was small, and our study power for investigating low-incidence outcomes such as mortality was inadequate. Third, the outcomes we assessed have subjective components in their nature. Finally, synbiotics and probiotics are available in various compositions and doses, so our results cannot be generalized to other types.

## 5. Conclusion

According to our findings, prescribing multi-strain synbiotics reduces the incidence of NEC in VLBW infants. These synbiotics also promote better breastfeeding tolerance and enhance the weight gain rate in these infants. Furthermore, their administration correlates with reduced hospital stay durations for these newborns. Therefore, clinicians may consider recommending synbiotics for VLBW neonates. Nevertheless, additional research is essential to fully assess their efficacy and safety before implementing them widely for NEC prevention. It is also crucial to investigate each component’s specific mechanisms of action.

## Acknowledgments

We thank the parents of the neonates, the nurses, the residents of Imam Reza Hospital in Kermanshah, and the Imam Reza Hospital Research Center for their cooperation in conducting this study.

## Author contributions

**Conceptualization:** Homa Babaei, Amir Shahidolahi.

**Formal analysis:** Amir Ali Mahboobipour.

**Investigation:** Amir Shahidolahi.

**Supervision:** Homa Babaei.

**Writing – original draft:** Amir Ali Mahboobipour, Ali Bitaraf, Pourang Mohammadi, Mina Khosravifar.

**Writing – review & editing:** Amir Ali Mahboobipour, Ali Bitaraf, Homa Babaei.
